# Evaluation of Job-Related Anxiety Symptoms Among Brazilian Social Security Medical Experts

**DOI:** 10.6061/clinics/2018/e428

**Published:** 2018-10-10

**Authors:** João Guilherme Tavares Marchiori, Fabio P Saraiva, Liliane C G da Silva, Jessica B Garcia, Juliano C M Pina

**Affiliations:** I Programa de Pos-Graduacao em Medicina, Mestrado Profissional em Medicina, Universidade Federal do Espirito Santo, Vitoria, ES, BR; II Departamento de Medicina Especializada, Centro de Ciencias da Saude, Universidade Federal do Espirito Santo, Vitoria, ES, BR; III Departamento de Clinica Medica, Programa de Psiquiatria, Centro de Ciencias da Saude, Universidade Federal do Espirito Santo, Vitoria, ES, BR; IV Curso de Graduacao em Medicina, Escola Superior de Ciencias da Santa Casa de Vitoria, Vitoria, ES, BR; V Associacao Nacional de Medicina do Trabalho, Vitoria, ES, BR

**Keywords:** Insurance, Physician Services, Psychological Stress, Occupational Diseases, Burnout, Professional Anxiety

## Abstract

**OBJECTIVES:**

The function of a medical expert within the scope of the Brazilian social security system consists of medically evaluating the examinee to draw a conclusive opinion on the person's working ability capacity and to characterize the disability for social security and assistance purposes. Forensic decisions inevitably converge on two opposite outcomes: concession or refusal. Rejection is against the interests of the candidates, who can direct their disappointment and dissatisfaction at the professional, exposing the latter to potentially stressful situations. The present study aimed to determine whether the prevalence of stress and anxiety symptoms is higher among social security medical experts than among medical experts in other public service careers. The study was also intended to propose organizational changes aimed at the preservation and recovery of the mental health of medical experts.

**METHODS:**

The evaluation was conducted using a specific, previously validated job-related stress questionnaire and a series of questions about subjective perceptions of work performance, labor stressors and emotional status.

**RESULTS:**

We found an overwhelming and alarming prevalence of stress and dissatisfaction related to occupational aspects among social security experts, often culminating in emotional exhaustion, a characteristic feature of burnout syndrome.

**CONCLUSION:**

There is a high prevalence of job-related stress among social security doctors in Brazil, warranting implementation of specific measures to ensure the adequate provision of social security services to the population, thus avoiding social injustice and financial damage.

## INTRODUCTION

The Brazilian National Social Security Institute is the agency responsible for the provision of public health services, social assistance and pecuniary benefits to protect workers and their families. The granting of those benefits almost always depends on the discretion of an expert, culminating in a conclusion on the candidate's capacity for work and a characterization of the person's disability for public insurance reimbursements and social assistance purposes 1. The traditional paradigm of the diagnosis, prevention and treatment of diseases, while inherent to medical assistance, does not apply to expert duty. Instead, the expert evaluates the repercussions of previously diagnosed disease or injury on the laborer's working capacity. The focus is on clarifying legal implications and especially on the recognition of rights and the establishment of causal relationships. It is up to the social security medical experts (SSMEs) to decide whether there is an impairment of working capacity and whether such impairment results in partial or total incapacity, temporary or permanent, for all activities or for one or some specific duties 2,3. In addition to all these functions, SSMEs analyze the cost and legitimacy of conceding social security benefits arising from functional or anatomical sequelae, tax exemptions due to debilitating diseases, pregnancy-related accommodations, characterization of disability for special retirement purposes and social assistance benefits for those in extreme poverty and unfit for acts of civil life 4-6. The importance of social security in Brazil can be outlined by the number of grants conceded. In 2015 alone, the number of grants for temporary disability from disease total 15,588,262, with a cost of almost 19 billion reals 6.

This wide range of assignments has the potential to cause work overload, stress, anxiety and fatigue, as documented among professionals in other careers, with consequences that affect not only the professionals but also their families and patients 7-9. In addition to the multiplicity of assignments, which in itself is already a possible factor generating occupational stress, decisions that do not match the examinee's expectations eventually generate situations of verbal and physical violence, producing harmful and potentially serious psychological impacts 10,11.

In addition to the cumulative effect of hostility against SSMEs, the media have reported several instances of acute violence of large proportions and immediate import 12-16. The possible underreporting of violence must also be considered, as is often the case in health services 17-19. Underreporting by the victims, whether for fear of retaliation, lack of support from the institution or a desire to avoid reliving unpleasant situations, causes repression of negative anxieties and emotions, which potentially contributes to the aggravation of stress and the development of mental illness in the long term 10,11.

Considering the economic magnitude of public insurance concessions 20 and the consideration that impartiality, which is essential to expert activity, can be affected by psychiatric disorders arising from threats and other forms of violence, it is essential to monitor public experts' mental health to ensure the quality of this essential service 21-23.

The main objective of this study is to identify the prevalence of anxiety symptoms in SSMEs, compared to medical experts in other public careers. In addition, the study is intended to analyze the degree of correlation between a work stress assessment questionnaire (WSAQ) and subjective perceptions of stress and anxiety. Finally, we were able to craft some specific recommendations to attenuate the impact of occupational stressors on the health of public experts.

## MATERIALS AND METHODS

In this cross-sectional study, two comparison groups were created, one consisting of the SSMEs of the National Social Security Institute (Investigation Group (IG)) and the other consisting of publicly employed medical experts in other careers (Comparison Group). The Comparison Group (CG) encompassed experts from the Institute for Social Security of Espírito Santo State Employees (IPAJM) and other locally based government facilities that perform expert assessment of federal civil employees, known as SIASS (Integrated Subsystem of Attention to Civil Servants' Health). An informed consent document was provided to, read by and signed by all participants.

Participants were given a validated WSAQ 24 (Appendix 1), as well as 9 questions about their subjective perceptions of stress, work environment, career, future professional prospects and evolution of motivation and competence over time. The complete research form, called the Medical Experts' Stress Questionnaire (MESQ), was applied between February and June 2017 and can be found in Appendix 1. The WSAQ is composed of 23 statements to be rated on a scale from 1 to 5, depending on the level of agreement. A respondent's situation was considered stressful if the arithmetic mean was greater than or equal to 2.5 24. The evaluation proceeds to items 2 and 3 of the MESQ, which address professional activities and work environments, respectively. Questions 4 to 8 surveyed the temporal evolution of stress or anxiety, motivation, competence and career success and were intended to assess whether negative psychological factors caused impacts or losses to users of the social security system. More specifically, question 6 sought indications of significant emotional impairment still controlled by internal mechanisms (second response option), or even marked emotional exhaustion (third response option), which could support the diagnosis of burnout.

Of the 74 SSMEs active in the State of Espírito Santo, Brazil, 65 returned surveys. In the CG, we obtained responses from 21 of the 24 physicians working at IPAJM and SIASS. Those who worked simultaneously for Social Security and SIASS were excluded from the study.

The identified variables were submitted to statistical analysis using Student's t-test, the chi-squared test, the Mann-Whitney nonparametric test and Fisher's exact test 25. The weekly number of medical exams was analyzed by the nonparametric Mann-Whitney Test, as were all situations reported on the WSAQ. Fisher's exact test and the chi-squared test were applied to subjective perceptions of the work routine and the work environment, considerations on loss of motivation, desire to change career or intention to give up, reduction of competence and career success perceptions. Chi-squared tests were utilized to assess the evolving states of mood, anxiety, stress and emotional exhaustion. The software used for calculation was SPSS 17.0 for Windows, and *p*-values less than or equal to 0.05 were considered statistically significant for all variables.

Initially, all variables were analyzed descriptively. For quantitative variables, the analysis process was performed by observing the minimum and maximum values and calculating the means, standard deviations and medians. For qualitative variables, relative and absolute frequencies were calculated. Student's t-test was used to compare the mean ages of the two groups. Score comparisons between pairs of groups were performed using the nonparametric Mann-Whitney test 25, and comparisons among three groups used the Kruskal-Wallis nonparametric test, as the assumption of data normality was rejected. The chi-squared test or Fisher's exact test was used to test for homogeneity between proportions. The study was approved by the local research ethics boards and the directors of the public agencies involved.

## RESULTS

The present study showed elevated overall WSAQ scores among the SSMEs, indicating that, in general, these professionals are more anxious than those in the CG. The subjective perceptions of stress, anxiety, demotivation, reduction of competence, harm to users of social security services, regret and intention to abandon their career were also more present among social security experts than among controls. The demographic variables, the means of transportation used for commuting and the time spent commuting did not differ between the groups. It was observed that the SSMEs had longer career experience than the controls, with 84.6% of SSMEs having worked for 5 years or more, compared to only 52.4% in the CG. In the IG, no participant had been on duty for less than 3 years, compared to 42.9% in the CG. Regarding the number of weekly medical exams, we observed that the SSMEs performed, on average, 62.09 tests compared to 24.1 by other experts, with *p*<0.001 (Table 1).

On analysis of each individual topic of the WSAQ, the SSMEs showed significantly higher scores regarding the nervousness caused by the distribution of tasks and insufficient time to complete the existing workload; irritation due to the type of control present at work; distress caused by lack of professional autonomy; irritation as a consequence of poor disclosure of organizational decisions; annoyance caused by inadequate treatment by or poor communication with the hierarchical superior; need to perform tasks above or below individual capacity and lack of professional training and updating; bad mood caused by many hours of work and isolation within the organization; and irritation related to unfairness in promotions in the work environment and low valuation by superiors and to the distress caused by poor prospects for career growth.

Moving on to the analysis of subjective, unquantifiable perceptions regarding usual activities and working environments, we again found differences between the groups. In the IG compared to the CG, there was increased classification of activities as tiresome and stressful and of the work environment as unsafe, uncomfortable and oppressive. On the other hand, the CG predominantly rated their workplace as safe, comfortable and free, providing full professional autonomy (Table 2).

Regarding WSAQ questions 4 to 8, there was a significant difference in the temporal evolution of the mood state. Among the SSMEs, 69.2% reported that they felt more cheerful at the beginning of their careers than at present, compared to 19.1% in the CG (Table 3). In addition, only 20% of the SSMEs reported that their mood state did not change over time, compared to 61.8% of the experts from the CG. The perception of anxiety also worsened in 64.6% of SSMEs versus 17.3% in the other experts. SSMEs reported that they were emotionally affected or very emotionally worn in 73.8% of cases, while only 14.3% of CG experts said they were somewhat shaken, and none of the CG experts considered themselves too worn out. The sensation of reduced success and competence, addressed in question 8 of the WSAQ, although more prevalent in the SSMEs than in the CG, did not reach a statistically significant difference between the groups (Table 4). As for regrets about having pursued the career and the intention to leave it, the IG presented a higher prevalence of affirmative answers than the CG.

When verifying the concordance of the WSAQ scores with the subjective perception of anxiety, we found that 83.33% of the SSMEs deemed anxious by the WSAQ also stated that they felt more anxious in the present than at the beginning of their career. Moreover, of those who were not rated as anxious by the WSAQ, only 41.38% declared themselves more anxious now than in the past. These findings were statistically significant (*p*<0.001) by the chi-squared test. The situation was similar in the CG, where 100% of the participants labeled anxious by the WSAQ also considered themselves more anxious now than in the past, while among those who did not meet the criteria for anxiety according to the WSAQ, only 1 expert (5.56%) declared himself subjectively anxious.

## DISCUSSION

SSMEs' mental health is permanently affected by work organization, safety, autonomy and workload issues. The marked differences between the SSME and non-SSME groups reflect a reality that has long been perceived by professionals working in the Brazilian social security system but not demonstrated by scientific means. Many stress variables were found to have increased intensity among SSMEs and were not mutually exclusive, behaving, in fact, as synergistic actors for the production of anxiety and occupational stress symptoms.

There was a marked excess of weekly medical exams assigned to the SSMEs (Table 1), which raises the hypothesis of a causal relation between a heavy workload and the development of stress symptoms. In this sense, a timely investigative approach would be the analytical study of cut-offs beyond which further exams would render doctors prone to stress development.

The SSMEs showed significantly higher scores than the non-SSMEs on several WSAQ topics. Such scores objectively express adverse organizational situations, such as the inadequate or inhomogeneous distribution of tasks and the multiplicity of assignments, which can be exemplified as evaluation of working capacity, recognition of serious illnesses for tax benefits, granting of subsistence benefits to persons in extreme poverty and social vulnerability, analysis of sequelae for compensation benefits, pensions to adults unfit for the duties of civilian life, maternity compensation, analysis and granting of disability retirement and special retirement, professional rehabilitation and reinsertion in the labor market, characterization of deficiencies for the purpose of anticipating retirement, and attending to various judicial demands 2-6.

In this unfavorable scenario of excessive professional assignments, it is difficult from a management perspective to distribute such tasks uniformly to the laborers. As a consequence, some are more intensively subjected to complex or time-consuming tasks, while others are assigned more stressful, more uncertain, more bothersome, or otherwise less desirable tasks. This nonuniformity would explain the negative impact of the inadequate task distribution pointed out in the WSAQ. The multiplicity of tasks, the innumerable recursive pathways, and the freedom for users to schedule medical exams in an unlimited and repeated manner 4 contribute to the establishment of a redundant and excessive workload, also perceived by the experts as an inciting factor of anxiety.

In order to correct such excesses, we recommend multisectoral measures, such as de-bureaucratization, limitation of recursive pathways and respect for the experts' autonomy in their decisions and competences. Organizational decisions are often carried out through normative directions and instructions, memoranda, decrees, and laws. Keeping completely updated is virtually impossible, given the already existent workload. SSMEs must simultaneously address the constant demand for legislative and medical updates, which probably contributes to the subjective perception of overload and stress. This reality is externalized by means of significantly elevated scores related to deficiency in the dissemination of organizational decisions and training for professional qualification. Indeed, it would be interesting to spread the central content of normative changes through constant training, with practical and usual situations, combined with proper continued medical education programs.

In reference to the questionnaire, problems were detected in the network of interpersonal relationships, with negative perceptions about pressures, lack of recognition, undervaluation and bad treatment by hierarchical superiors. The nature of the tasks assigned to the professionals by their managers, whether above or below their cognitive abilities, may represent a risk factor for increased anxiety symptoms as well as a perception of favoritism devoid of merit among coworkers 26. Negative considerations of workers towards their superiors have been identified and can now be addressed with proper strategies.

Individual perceptions regarding professional activities and work environments (Table 2) undoubtedly exposed the antagonistic situation experienced by the two groups. Thus, it is fully conceivable that stressful activities and insecure, uncomfortable and oppressive environments may generate anxiety and stress. Therefore, it is essential to propose interventions aimed at improving safety, ergonomics and autonomy as integrated solutions to reduce anxiety and stress. Special mention should be given to the oppression reported by many SSMEs. It is known that the internal courts of appeal, which have deliberative power, are composed of several nonmedical professionals. In other words, appeals are judged, albeit not exclusively, by professionals not graduated in the medical field. Thus, any changes in forensic decisions may be perceived negatively by experts as a violation of their freedoms and competences.

We found that the passage of time negatively affected the participants' mood, such that 23% of the SSMEs (15 experts) were already very emotionally worn out, and 60% of that subset (9 experts) even believed that their emotional impairment could have caused harm to users. The remarkable emotional exhaustion and the possibility of injury to third parties are two essential components for the diagnosis of burnout 27.

Stressful routines are known risk factors for the development of anxiety and mood disorders, especially in dealing with a population who are both clients and patients 27, and these factors outline a reasonable scenario of depression arising from chronic stress, ultimately confirming that mental disorders commonly have causal relationships with work practices 27. Changes should be guided by the identification of occupational risk factors that are known to affect mental health. The main factors are insecurity, repetitiveness and monotony of tasks, lack of autonomy and control, imbalance between effort and reward (not only income but also satisfaction upon completion of important tasks), lack of support from the institution and a sense of injustice or unfair favoritism 28. A large British study on the health status of the economically active population has adopted the perspective that health and well-being at work not only reduce absenteeism but also improve productivity and profitability and, therefore, should be always promoted, although such measures require high investments from employers 28. In the social security system, productivity and profitability could be better called economy and efficiency, two of the fundamental principles with which employees should carry out their public assignments. Although there are many particularities of each organizational environment and productive sector, there are six major stressors (demands, control, support, relationships, skills/assignments and organizational changes) that can be taken into account to tailor a specific strategy for any work environment 29.

Returning to the SSMEs' particular situation, a strategic plan should address specific stressors facing workers in the social security environment, identified by analysis of WSAQ responses and Table 3. Thus, the sense of insecurity can be ameliorated by an increase in the number and training of security personnel; by the establishment of architectural adaptations to provide escape routes and physically separate the main circulation areas between laborers and the public; and by the installation of panic buttons and other security devices. Lack of autonomy, in turn, must be treated by increasing the experts' freedom in their decisions, eliminating instances of inappropriate interference by management. Improvements in professional qualification are another aspect to be addressed because trained professionals feel more secure in their decisions, which may play a role in stress prevention. It is also necessary to implement preventive health promotion programs, as well as strategies aimed at early identification of mental illness, to avoid harm to laborers, social security employees and citizens.

The development of support systems for the experts' mental health must follow a step-by-step approach, involving research, consulting, and active participation of employees, to enable the identification of risk factors, their causes, and methods of prevention with the consequent establishment of goals, objectives and outcome assessment tools.

Although stressful situations can be minimized through the proposed approach, it is virtually impossible to create work environments that are completely armored against all stress-generating factors because public and private institutions always deal, directly or indirectly, with goals, deadlines, responsibilities and conflicts of interest. Thus, it is essential that experts be offered adequate psychological support aimed at the development and improvement of resilience.

The present study also verified the level of agreement between the WSAQ score and the subjective perception of anxiety. In the IG, 83.3% of those who scored at or above 2.5 also said they were more anxious now than in the past. An analogous situation was observed in the CG, in which 100% of those classified as anxious by the WSAQ were also considered anxious from a subjective perspective. Thus, there is good agreement between the objective criteria used (scores) and the experience and subjective perception of anxiety by the medical experts.

The last two MESQ questions aimed to identify those who, due to stress, anxiety and other work-related negative perceptions, were willing to give up their ambitions or migrate to a more attractive career. In other words, they sought, implicitly, to determine whether negative psychological factors could lead the experts to no longer prioritize the struggle for career enhancement but rather to abandon it. The lack of interest in attaining or striving for improvements may represent not only a more advanced stage of psychological involvement but also a greater probability of pathological affective distancing, a typical characteristic of burnout 30. Again, the SSME group showed greater intentions to leave their careers and a greater degree of regret than the control group did. Such findings help define the severity of the situation because distancing, disgust, demotivation and regret are negative psychological factors related to the development of burnout 30.

Although it is clear that SSMEs are inserted in a highly unhealthy work context, the present study has some limitations, the main ones being the small size of the CG and the impossibility of testing associative hypotheses, a fact inherent to its cross-sectional design. Nevertheless, this study allows the formulation of several hypotheses of causality between specific aspects of the expert work environment and the development of stress symptoms. Such hypotheses can be tested in either observational or interventional analytical studies in the future, complementing and assigning the weight of each factor in the triggering of stress, and at the same time controlling potential confounding variables. Even with methodological limitations, the association between inadequate working conditions for SSMEs and the increased presence of stress symptoms is clear. Another limitation may be the differences in career duration. It was observed that the SSMEs had longer career experience than the non-SSMEs, with 84.6% of the former and only 52.4% of the latter having worked for 5 years or more. In the SSME group, no participants had been on duty for less than 3 years, as opposed to 42.9% in the GC. These findings could influence the incidence and severity of stress. We hypothesize that longer careers can be a protective factor against stress, since it allows the improvement of adaptation and resilience techniques, or even a causal factor, if these same defense mechanisms do not develop adequately at the individual level. There is also the possibility that career length does not influence the incidence or severity stress. This issue could be better addressed in studies where analytical methods allow proper control of all variables.

In summary, SSMEs showed alarmingly elevated levels of stress, demotivation and performance impairment in comparison to the other medical experts evaluated. The reasons for such findings include organizational and administrative issues, such as excessive workload, multiplicity of assignments, insecurity, lack of institutional support, training programs, professional autonomy and ergonomic inadequacies. Based on these findings, we were able to formulate recommendations encompassing the six major occupational stressors 29. In this way, in regard to the “demands” issue, there must be an effort to distribute tasks homogeneously, which prevents work monotony and complete domination of the workload by stressful tasks. Concerning the “control” issue, we recommend reinforcing the autonomy and freedom of the experts' decisions. For the “support” and “relationships” questions, it would be worthwhile to implement preventive health promotion programs and mental illness early detection programs, as well as group dynamics aiming at motivation and development of psychological techniques to cope with stress. With regard to the topics of “skills” and “assignments”, it is important to implement continued medical education programs, focused on real case discussions and provide constant updates on laws and official normative instructions. Finally, “organization” issues can be addressed by means of improvements in workplace security, limitation of recursive pathways, de-bureaucratization and protection of experts against verbal or physical aggression. These changes can work as protective measures against occupational stress, ultimately improving efficiency; as a consequence, they have the potential to avoid harm to the public finances and the beneficiaries of the Brazilian Social Security services.

## AUTHOR CONTRIBUTIONS

Marchiori JG participated in the conception and planning of the study, was responsible for the analysis, acquisition and interpretation of the data, manuscript writing, approved the manuscript final version to be published and agreed to be accountable for all aspects of the work in ensuring that questions related to the accuracy or integrity of any of its parts were appropriately investigated and resolved. Saraiva FP participated in the conception of the study, acquisition and interpretation of the data, manuscript writing, approved the manuscript final version to be published and agreed to be accountable for all aspects of the work in ensuring that questions related to the accuracy or integrity of any part of its parts were appropriately investigated and resolved. Silva LC participated in the conception of the study, interpretation of the data and in critically revision of the manuscript for important intellectual content, approved the manuscript final version to be published and agreed to be accountable for all aspects of the work in ensuring that questions related to the accuracy or integrity of any of its parts were appropriately investigated and resolved. Garcia JB participated in acquiring the data and manuscript writing the work, approved the manuscript final version to be published and agreed to be accountable for all aspects of the work in ensuring that questions related to the accuracy or integrity of any of its parts were appropriately investigated and resolved. Pina JC participated in acquiring the data and manuscript writing, approved the manuscript final version to be published and agreed to be accountable for all aspects of the work in ensuring that questions related to the accuracy or integrity of any of its parts were appropriately investigated and resolved.

## Appendix 1

**Table:** 

Work Stress Assessment Questionnaire (first part of Medical Experts' Stress Questionnaire)						
Role:	Age	Gender (M/F) ()				
Work Institution:	Marital Status:	Number of Medical Exams per Week				
Length of Commute to Work (min):		Preferred Means of Transportation:				
Career Length (yr)	<1 ( )	1-3 ( )	3-5 ( )	5-10 ( )	>10 ( )	
1) Below are listed several situations that can occur in your work routine. Please read each statement and use the scale below to give your opinion on each one:						
1: Strongly Disagree	2: Disagree	3: Partially Agree	4: Agree	5: Strongly Agree		
		1	2	3	4	5
1A	The way tasks are distributed in my area has made me nervous	( )	( )	( )	( )	( )
1B	The type of control in my work irritates me	( )	( )	( )	( )	( )
1C	The lack of autonomy in the execution of my work has been exhausting	( )	( )	( )	( )	( )
1D	I have been uncomfortable with my superior's lack of confidence in my work	( )	( )	( )	( )	( )
1E	I am irritated by the lack of information on organizational decisions	( )	( )	( )	( )	( )
1F	I feel bothered by the lack of information about my tasks at work	( )	( )	( )	( )	( )
1G	The lack of communication between me and my co-workers makes me angry	( )	( )	( )	( )	( )
1H	I feel bothered by my superior treating me badly in front of co-workers	( )	( )	( )	( )	( )
1I	I feel uncomfortable having to perform tasks that are beyond my capacity	( )	( )	( )	( )	( )
1J	I am in a bad mood about having to work for many hours	( )	( )	( )	( )	( )
1K	I feel uncomfortable with the communication between me and my superior	( )	( )	( )	( )	( )
1L	I am irritated with discrimination/favoritism in my work environment	( )	( )	( )	( )	( )
1M	I have been bothered by the lack of professional training offered	( )	( )	( )	( )	( )
1N	I am in a bad mood because I feel isolated in the organization	( )	( )	( )	( )	( )
1O	I get annoyed at being undervalued by my superiors	( )	( )	( )	( )	( )
1P	The scarcity of prospects for career growth has made me distressed	( )	( )	( )	( )	( )
1Q	I have been bothered by working on tasks below my skill level	( )	( )	( )	( )	( )
1R	Competition in my work environment has left me in a bad mood	( )	( )	( )	( )	( )
1S	Lack of understanding of my responsibilities in this job has caused irritation	( )	( )	( )	( )	( )
1T	I have been nervous because my superior gave me contradictory orders	( )	( )	( )	( )	( )
1U	I feel annoyed by my superior hiding my successes from other people	( )	( )	( )	( )	( )
1V	Insufficient time to accomplish my workload makes me nervous	( )	( )	( )	( )	( )
1X	I am annoyed by my superior refusing to entrust me with important responsibilities	( )	( )	( )	( )	( )
Questions 2-9 (second part of Medical Experts' Stress Questionnaire).						
2) In general, how do you classify your usual activities at present? (More than one answer may be ticked.)						
( ) Motivating	( ) Frustrating	( ) Rewarding	( ) Monotonous, but without causing strong feelings of appreciation or disapproval			
( ) Tiring	( ) Stressing	( ) Monotonous and boring				
3) In general, how do you rate your work environment at present? (More than one answer may be ticked.)						
( ) Safe	( ) Unsafe	( ) Cozy or comfortable				
( ) Uncomfortable or anti-ergonomic	( ) Oppressive	( ) Free, allowing me to fully exercise my professional autonomy				
4) In general, do you feel changes in your mood when compared to the time you started your activities as an expert?						
( ) No, my mood has not changed over time.		( ) Yes, I felt more excited and in better spirits at first.		( ) Yes, I feel more excited and in better spirits now.		
5) In general, do you feel changes in your stress/anxiety level when compared to the beginning of your career?						
( ) No, my stress or anxiety level did not change over time.		( ) Yes, I am more stressed or anxious now than in the past.		( ) Yes, I feel less stressed or anxious now than in the past.		
6) How do you describe your current emotional state in relation to work?						
( ) My emotional situation is stable because I feel that my emotions are well adjusted to my assignments.						
( ) My emotions are a little affected because of my job conditions, but I am able to deal reasonably well with this.						
( ) I am very emotionally worn out.						
7) If you have felt loss of motivation with work, do you think that this may have caused harm to the recipients of your services or the institution where you work?						
( ) YES				( ) NO		
8) Do you feel that your competence or success at work has been affected over time?						
( ) YES, I feel that my competence and success have diminished				( ) NO		
9) If you could go back, would you have chosen another activity or career?						
( ) YES				( ) NO		
10) Do you intend to leave your career if there is another job opportunity with similar income and conditions?						
( ) YES				( ) NO		

## Figures and Tables

**Table 1:** Demographic data and weekly number of medical exams (n=86).

		SSME*		
	Total (n=86)	No (n=21)	Yes (n=65)	*p*
**Age**				
(Mean±SD)	46.98±10.12	48.48±12.71	46.49±9.19	0.438(1)
**Gender**				
Male	52 (60.5%)	16 (76.2%)	36 (55.4%)	0.090(2)
**Marital Status**				
Single	8 (9.3%)	2 (9.5%)	6 (9.2%)	0.109(4)
Married	71 (82.6%)	15 (71.4%)	56 (86.2%)	
Stable Union	1 (1.2%)	1 (4.8%)	0 (0.0%)	
Divorced	6 (6.9%)	3 (14.3%)	3 (4.6%)	
**Working Time (years)**				
Less than 1	3 (3.5%)	3 (14.3%)	0 (0.0%)	<0.001(4)
1 - 3	6 (6.9%)	6 (28.6%)	0 (0.0%)	
3 - 5	11 (12.8%)	1 (4.8%)	10 (15.4%)	
5 -10	27 (31.4%)	3 (14.3%)	24 (36.9%)	
More than 10	39 (45.4%)	8 (38.1%)	31 (47.7%)	
**Means of Transportation**				
Car	81 (94.2%)	20 (95.2%)	61 (93.9%)	0.763(4)
Bus	2 (2.3%)	0 (0.0%)	2 (3.1%)	
On Foot	2 (2.3%)	1 (4.8%)	1 (1.5%)	
Motorcycle	1 (1.2%)	0 (0.0%)	1 (1.5%)	
**Duration of Commute from Home to Work (minutes)**				
Mean±SD	25.58±33.25	17.00±10,07	28.35±37.48	0.155(3)
Median	15	15	20	
**Number of Exams**				
Mean±SD	52.81±26.68	24.10±18.25	62.09±21.99	<0.001(3)
Median	60	20	75	

*Social Security Medical Expert.

^(1)^Descriptive probability level according to Student’s t-test, ^(2)^ Descriptive probability level according to the chi-squared test, ^(3)^ Descriptive probability level according to the Mann-Whitney nonparametric test, ^(4)^ Descriptive probability level according to Fisher’s exact test.

**Table 2:** Absolute and relative distributions of usual activities and work environment, separated by study group.

						SSME*			
		Total (n=86)		No (n=21)		Yes (n=65)		
Variable	Classification	n	%	n	%	n	%	*p*
	Motivating	14	16.3	6	28.6	8	12.3	0.096(2)
	Tiresome	50	58.1	4	19.0	46	70.8	<0.001(1)
	Frustrating	16	18.6	1	4.8	15	23.1	0.103(2)
Usual Activities	Stressful	45	52.3	3	14.3	42	64.6	<0.001(1)
	Rewarding	13	15.1	5	23.8	8	12.3	0.291(2)
	Monotonous and boring	10	11.6	0	0.0	10	15.4	0.110(2)
	Monotonous	13	15.1	6	28.6	7	10.8	0.076(2)
	Safe	14	16.3	10	47.6	4	6.2	<0.001^(2)^
	Unsafe	55	64.0	3	14.3	52	80.0	<0.001(1)
Work Environment	Comfortable	12	14.0	8	38.1	4	6.2	<0.001(2)
	Uncomfortable	36	41.9	4	19.0	32	49.2	0.002(1)
	Oppressive	15	17.4	0	0.0	15	23.1	0.017(2)
	Free	15	17.4	9	42.9	6	9.2	0.001(2)

*Social Security Medical Expert.

^(1)^Descriptive probability level according to the chi-squared test, ^(2)^ Descriptive probability level according to Fisher’s exact test.

**Table 3:** Absolute and relative distributions of mood evolution, stress level and emotional status separated by study group.

						SSME*			
		Total (n=86)		No (n=21)		Yes (n=65)		
Variable	Classification	n	%	n	%	n	%	*p*(1)
Mood Changes over Time	No	26	30.2	13	61.8	13	20.0	<0.001
	Better mood in the past	49	57.0	4	19.1	45	69.2	
	Better mood now	11	12.8	4	19.1	7	10.8	
Stress/Anxiety Changes over Time	No	28	32.6	14	66.7	14	21.5	<0.001
	More stressed now	45	52.3	3	17.3	42	64.6	
	Less stressed now	13	15.1	4	19.0	9	13.9	
Current Emotional Status	Stable	35	40.7	18	85.7	17	26.2	<0.001
	A little shaken	36	41.9	3	14.3	33	50.8	
	Very worn	15	17.4	0	0.0	15	23.0	

*Social Security Medical Expert.

^(1)^Descriptive probability level according to the chi-squared test.

**Table 4:** Absolute and relative distributions of subjective performance perceptions and willingness to leave career.

					SSME*			
	Total (n=86)		No (n=21)		Yes (n=65)		
Variable	n	%	n	%	n	%	*p*
Harm to third parties	26	30.2	1	4.8	25	38.5	0.004(1)
Work success affected	15	17.4	1	4.8	14	21.5	0.103^(2)^
Would choose another career	35	41.2	2	9.5	33	51.6	<0.001(1)
Would abandon career	37	43.5	2	9.5	35	54.7	<0.001(1)

*Social Security Medical Expert.

^(1)^Descriptive probability level according to the chi-squared test, ^(2)^ Descriptive probability level according to Fisher’s exact test.
